# Supplementary material for: Magnetostatic twists in room-temperature skyrmions explored by nitrogen-vacancy center spin texture reconstruction
Source: Nat Commun. 2018 Jul 13;9:2712. doi: 10.1038/s41467-018-05158-9 (PMC6045603; doi:10.1038/s41467-018-05158-9)
Supplement: Supplementary file 1 — Supplementary Information [file 41467_2018_5158_MOESM1_ESM.pdf]

Supplementary Information:
Magnetostatic twists in room-temperature skyrmions explored by nitrogen-vacancy center spin-texture reconstruction

Y. Dovzhenko[†], F. Casola[†], S. Schlotter, T. X. Zhou,
F. Büttner, R. L. Walsworth, G. S. D. Beach, and A. Yacoby[*,]

[†] *These authors contributed equally to this work.*

[*] *Correspondence and requests for materials should be addressed to this author.*

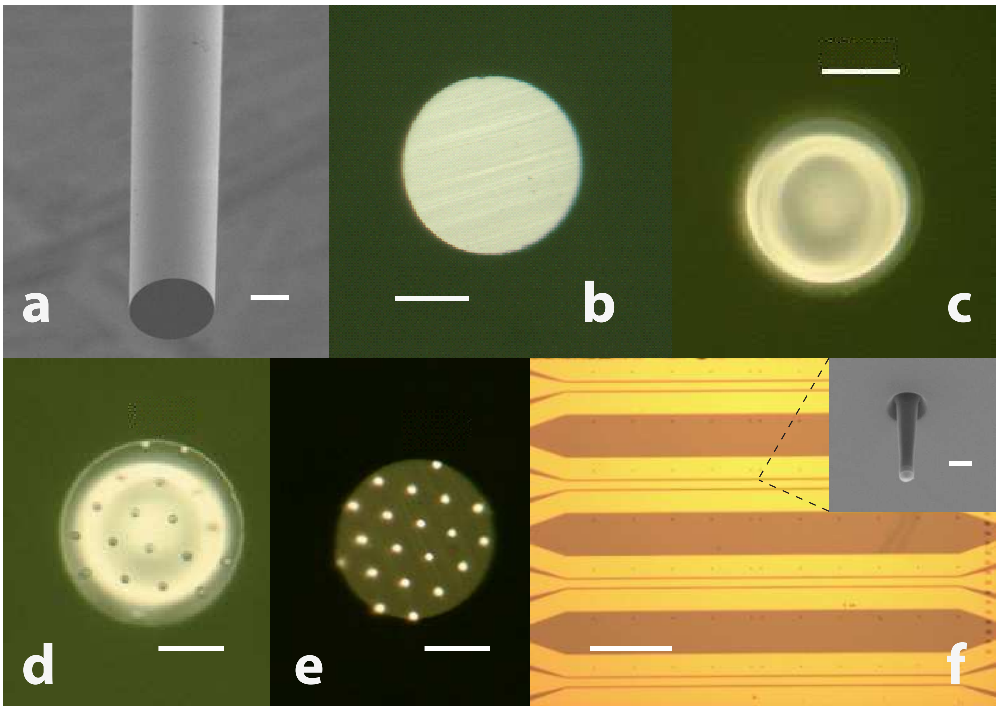

Supplementary Figure 1. **Fabrication of the tip with magnetic discs.** **a** Electron microscopy image of a pulled quartz fiber after the mechanical cleaving. **b** Optical microscopy image of the top surface of the tip, after being glued face-up on the aluminium holder. **c** The quartz tip after being spin coated with the PMMA resist. **d** A triangular lattice array of discs having a 2 μm diameter is defined using electron-beam lithography. **e** The tip after lift-off. The sputtered Pt/Co stacks are visible as highly reflective dots on the tip surface. **f** Four adjacent Ti:Au (5:100 nm) coplanar waveguides deposited via photolithography on the diamond surface. Inset: rows of diamond pillars are present within the gap between the waveguide plates. The scale bar is 20 μm for panels **a-e**, 500 μm (500 nm) for panel **f** (its inset).

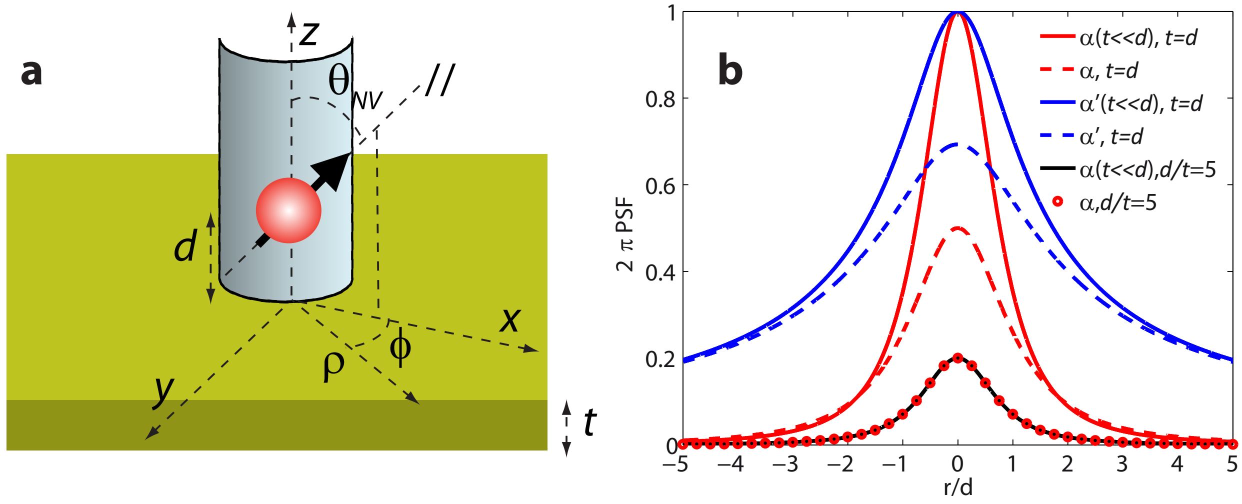

Supplementary Figure 2. Universal resolution functions for NV magnetometry. **a** Reference frame used for the calculations discussed in the text. The sketch represents an NV in a [100]-cut diamond pillar, with a quantization axis at an angle of $\theta_{\rm NV} = 54.7°$ with respect to the surface normal. The in-plane projection of the NV quantization axis is parallel to the x-axis. The NV has a distance d from the surface of the magnetic material and the magnetic film has a thickness t. **b** In the figure we plot the resolution functions derived in Supplementary Note 2 for various t/d ratios. Note that the resolution functions $\alpha_{x,y}$ and α_z have different units. In this plot the space dependence is shown with all the point spread functions (PSFs) plotted using $d = 1$. For the regime $t \ll d$ we use equations (11) and (13), while the full expression for $\alpha_{x,y}, \alpha_z$ is retained in all the other cases.

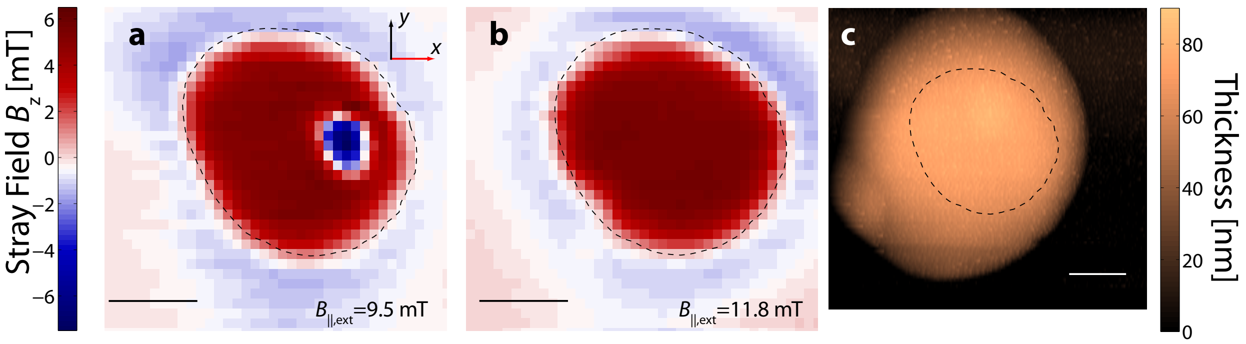

Supplementary Figure 3. **Calibration of the saturation magnetization and topography of the patterned disc. a** Reconstructed B_z stray field measured with a 9.5 mT bias field (see main text for details). The black dashed line outlines the boundary of the region within which the largest stray field is recorded **b** Same as in **a**, but with a bias field of 11.8 mT. The black dashed line is the same as in **a**. We clearly see that the bubble-like feature has disappeared at this field. Note also that the stray field B_z is qualitatively constant while moving along the boundary of the magnetic disc, supporting the assumptions that at these fields the magnetization is mostly out-of-plane. **c** Surface topography recorded by monitoring the piezo voltage V_z. The dashed black boundary is the same as in **a** and **b**, stressing the fact that the region in which we observe a magnetic stray field is smaller than the actual physical disc size. The scale bar is 500 nm for all panels.

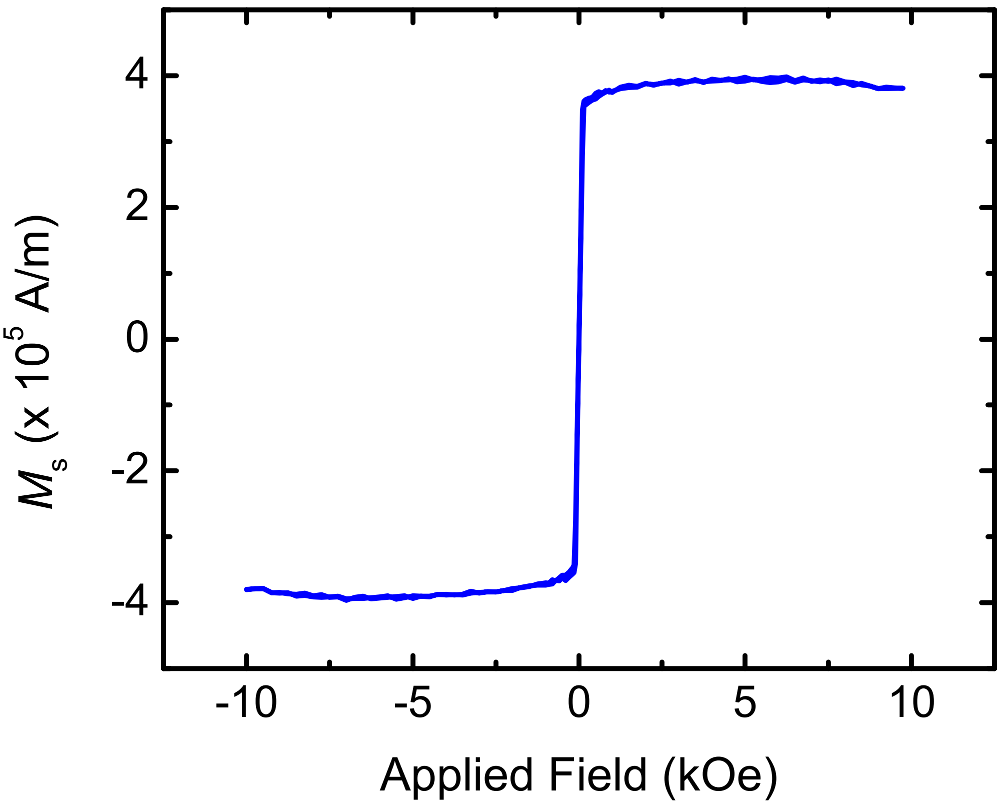

Supplementary Figure 4. Field-dependent magnetization of the reference Si substrate. Magnetization measured via Vibrating Sample Magnetometry (VSM) of a Si substrate held at the same height as the surface of the quartz tip during sputtering. The measurement reveals a bulk magnetization at saturation of the order of $M_s \simeq 3.8 \cdot 10^5$ A/m.

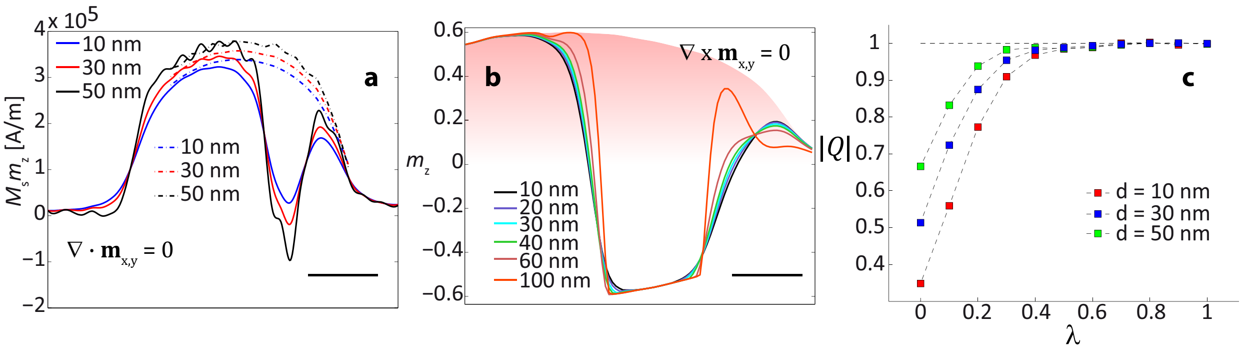

Supplementary Figure 5. **Stability of the solution upon varying the NV depth.a** Cut through the reconstructed out-of-plane magnetization in the Bloch gauge. The solid (dashed) lines represent solutions at the two different bias fields of 9.5 (11.8) mT. As the NV depth increases the noise in m_z also increases due to the exponential prefactor in eq. (7). Scale bar is 400 nm. **b** Cut through the skyrmion core displaying the reconstructed magnetic structure in the Néel gauge for different NV depths d (solid lines). As d increases the m_z profile near the skyrmion edges gets steeper in order to keep the resulting stray field constant. The scale bar is 200 nm. **c** Absolute value of the topological number for different NV depths, while tuning the spin configuration from Bloch to Néel with the parameter λ defined in eq. (40).

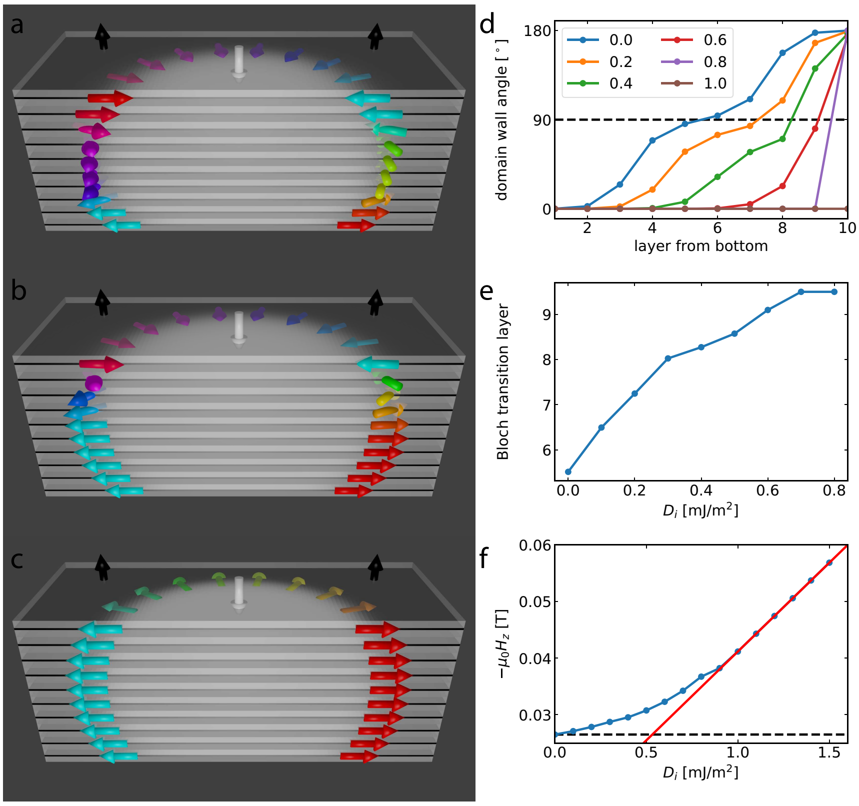

Supplementary Figure 6. **Helicity change along z in multilayers with different DMI. a-c** 3D cross section through the center of a skyrmion spin structure in the same multilayer as in Fig. 5 of the main text, with DMI values of $D_i = 0$ (**a**), $D_i = 0.5$ mJ/m^2 (**b**), and $D_i = 1.0$ mJ/m^2 (**c**). The colored arrows indicate the magnetization orientation in and position of the domain wall surrounding the skyrmion in each respective layer. The skyrmion radius is largest in the central layers. **d** Domain wall angle (here equivalent to the helicity) for the individual layers as a function of DMI, as indicated by the line color. The legend specifies the DMI value for each color in units of mJ/m^2. **e** Layer in which the domain wall angle is 90°, where fractional values are obtained by linear interpolation of the data in **d**. **f** Field required to stabilize a $R = 50$ nm radius skyrmion in the multilayer as a function of DMI. For large DMI, i.e., once all layers have a left-handed chirality, the field scales linearly with D_i, as indicated by the red line. For $D_i < 1$ mJ/m^2, the required field is larger than expected from a material with uniform magnetization along z.

Supplementary Note 1. Sample preparation

In our experiment, magnetic discs were deposited at the top of cleaved quartz fiber $\sim$ 50 μm in diameter. We employ Sutter Instruments quartz rods with the initial diameter of 1 mm. The fiber diameter is controlled via a laser-based puller (P-2000 Sutter Instruments). Fabrication proceeds with a controlled mechanical cleaving of the pulled fiber after the introduction of an intentional crack by means of a diamond scribe. The resulting tip, shown in Supplementary Figure 1a, is glued face-up (Supplementary Figure 1b) on an aluminum holder (not shown). The position of the tip with respect to markers present on the holder can be measured via electron microscopy prior to the application of the resist used for lithography. The holder is subsequently mechanically mounted on a conventional spinner disc. We applied on the free-standing tips a few drops of a Microchem C6 PMMA resist. After spinning, the deposited resist (see Supplementary Figure 1c) is exposed via electron-beam lithography with a triangular lattice array of discs having a 2 μm diameter (Supplementary Figure 1d). We then deposited via sputtering a Ta(3 nm)/[Pt(3 nm)/Co(1.1 nm)/Ta(4 nm)]x10 stack. The bottom Ta layer is deposited as a seed layer to increase sample adhesion and enhance perpendicular magnetic anisotropy. The metal layers are deposited by d.c. magnetron sputtering at 2 mTorr Ar (Ta) and 3 mTorr Ar (Pt, Co), with a background pressure of $5 \cdot 10^{-6}$ Torr. Deposition rates are < 0.1 nm s^{-1} and calibrated by X-ray reflectivity. Reference Si substrates are held at the same height as the surface of the quartz tips and used to calibrate the saturation magnetization of the material (see also Supplementary Note 6). Subsequent immersion into acetone of the tip and the holder dissolves the glue and the resist, leaving the tip in the final state shown in Supplementary Figure 1e, ready to be glued onto the tuning fork used for the experiment. Our experiments were performed with a type IIa diamond grown by chemical vapor deposition by Element 6 measuring 4x2x0.05 mm^3. We studied NV centres formed by ^{15}N ion implantation at an energy of 18 keV and a density of 500/μm^2 and subsequent annealing for 2 hours at 800°C. This implantation energy is expected to yield NV centres at an estimated $\sim$30 nm depth from the diamond surface[1].

For the diamond pillars we first prepared an etch mask, patterned on the diamond via electron beam lithography using a FOX 16 flowable oxide resist from Dow Corning. Adhesion of the resist was guaranteed by a very thin, $\sim$ 10 nm layer of Titanium deposited via electron beam evaporation. The exposed etch mask pattern was transferred onto the diamond via a conventional top down anisotropic plasma etch performed in a Unaxis Shuttleline ICP reactive ion etching (RIE) system. An initial Ar/Cl_2 plasma etch was used to remove the Titanium adhesion layer, while O_2 plasma was used to etch the diamond. A typical $\sim$ 1.5 μm tall and $\sim$ 200 nm wide diamond

pillar, imaged via electron microscopy, is shown in the inset of Supplementary Figure 1f. We then defined a set of Ti:Au (5:100 nm) coplanar waveguides via photolithography. The gaps between the central conductor and the ground plate of the waveguides were aligned (see Supplementary Figure 1f) with the pillar rows via alignment markers defined on the diamond during the same O_2 etching described above.

Supplementary Note 2. Principles of stray field magnetometry

Because of the spatial confinement of its local spin density, to a volume below 1 nm^3 (see Ref. 2), a nitrogen-vacancy centre in diamond can be well approximated as a point-like sensor of magnetic fields.

Following other works[3,4], we will consider the following Hamiltonian for this spin-1 defect:

$$\mathscr{H} = D(\hat{S}_{\|})^2 + \gamma B_{\|}\hat{S}_{\|} + \gamma B_{\perp}\hat{S_{\perp}}, \tag{1}$$

where D is the zero-field splitting, $\gamma = 2.8025$ MHz/G is the NV gyromagnetic ratio and $\|, \perp$ indicate the directions parallel and perpendicular to the spin quantization axis of the colour centre. In this work, our [100]-cut diamond hosts NV centres forming an angle $\theta_{\mathrm{NV}} = \arccos(1/\sqrt{3})$ with the surface normal, defining the $\|$ direction (see also Supplementary Figure 2).

The value for D was obtained from a measurement of the electron-spin resonance (ESR) line splitting at small applied fields and was found to be $D = 2.8710(1)$ GHz.

In our experiments, both the upper (ω_+) and lower (ω_-) NV resonance frequency was measured at each point in space. The values for $B_{\|}$ and $B_{\perp}$ were then obtained using the following expressions[4]:

$$B_{\|} = \frac{\sqrt{-(D+\omega_+ - 2\omega_-)(D+\omega_- - 2\omega_+)(D+\omega_- + \omega_+)}}{3\gamma\sqrt{3D}}, \tag{2}$$

$$B_{\perp} = \frac{\sqrt{-(2D-\omega_+ - \omega_-)(2D+2\omega_- - \omega_+)(2D-\omega_- + 2\omega_+)}}{3\gamma\sqrt{3D}}. \tag{3}$$

In our experiment we measure the stray field $B_{\|}(x, y)$ in a plane at a distance d from the magnetic surface. We can call such quantity $B_{\|}(\boldsymbol{\rho}, d)$, with $\boldsymbol{\rho} = (x, y)$.

At the probe position the stray field is curl free and it is therefore possible to define a magnetostatic potential ϕ_{M} such that the vector field can be written as[5,6]:

$$\mathbf{B} = -\nabla\phi_{\mathrm{M}}(x, y). \tag{4}$$

As pointed out in the past[5,7], the previous relation implies that the stray field components are not independent. We will now derive the relation among the different stray field components and obtain further insight by starting from the following 2D Fourier transform of the quantity $\mathbf{B}(\boldsymbol{\rho}, d)$, defined in Ref. 4 as:

$$\mathbf{B}(\mathbf{k}, d) = \int_0^{2\pi}\int_0^{\infty} \mathbf{B}(\boldsymbol{\rho}, d) e^{-ik\rho\cos(\phi-\phi_k)} \rho \mathrm{d}\rho \mathrm{d}\phi. \tag{5}$$

The vectors $\mathbf{k}$ and $\boldsymbol{\rho}$ are 2-dimensional vectors in reciprocal and real space, forming an angle ϕ_k and ϕ with the x-axis (see also left of Supplementary Figure 2). As derived in Ref. 4, when the

stray field is produced by a sheet of magnetic dipoles distributed over a thickness t and with local magnetization $M_s\mathbf{m}(\boldsymbol{\rho}_j) = M_s[m_x(\boldsymbol{\rho}_j), m_y(\boldsymbol{\rho}_j), m_z(\boldsymbol{\rho}_j)]$, the stray field can be written in momentum space as[4]:

$$\mathbf{B}(\mathbf{k}, d) = \mathbf{D}(\mathbf{k}, d)\mathbf{m}(\mathbf{k}), \tag{6}$$

where the expression for the traceless symmetric $\mathscr{D}(\mathbf{k}, d)$ kernel matrix reads:

$$\mathbf{D}(\mathbf{k}, d) = \frac{\mu_0 M_s}{2}(e^{-dk} - e^{-(d+t)k}) \begin{pmatrix} -\cos^2(\phi_k) & -\frac{\sin(2\phi_k)}{2} & -i\cos(\phi_k) \\ -\frac{\sin(2\phi_k)}{2} & -\sin^2(\phi_k) & -i\sin(\phi_k) \\ -i\cos(\phi_k) & -i\sin(\phi_k) & 1 \end{pmatrix}. \tag{7}$$

Note that with respect to Ref. 4, we have included the finite size film thickness t by assuming the local magnetization vector $\mathbf{m}(\boldsymbol{\rho}_j)$ to be constant through the magnetic film thickness and therefore integrating that dimension out. M_s is the nominal, space-independent, saturation magnetization of the magnetic film.

From eq. (7) we can immediately realize that the rows of the matrix $\mathbf{D}(\mathbf{k}, d)$ are not independent and for this reason $\mathbf{D}(\mathbf{k}, d)$ is not invertible. In general, in eq. (6) it is impossible to obtain $\mathbf{m}$ by simply measuring all the components of the vector $\mathbf{B}$.

In momentum space, the algorithm relating the stray field component along the z-axis $B_z(\mathbf{k}, d)$ (see Supplementary Figure 2) to $B_{||}(\mathbf{k}, d)$, for an NV lying in the zx-plane, can be simply written as:

$$B_z(\mathbf{k}, d) = \frac{B_{||}(\mathbf{k}, d)}{\cos(\theta_{NV}) - i\sin(\theta_{NV})\cos(\phi_k)}, \tag{8}$$

In a similar way, all vector field components can be reconstructed without singularities from a single measurement of $B_{||}(\boldsymbol{\rho}, d)$ within a whole plane, provided $\theta_{NV} \neq \pi/2$.

Finally, we point out that the expression in eq. (7) includes also an analogy with the Huygens principle in optics. In particular, in order to reconstruct the field within a plane at a different distance $d' = d + h$ from the film, it will be simply enough to perform an inverse Fourier transform of the 2D Fourier transform at a distance d, multiplying by the prefactor $\exp(-kh)$. Such operations are known as upward or downward propagation for $h > 0$ or $h < 0$ and are well discussed in the literature[5].

Once B_z is known, all the other field components can be reconstructed according to (7). For instance, $B_x(\mathbf{k}, d) = -i\cos(\phi_k)B_z(\mathbf{k}, d)$. Using full knowledge of all the stray vector field components, in Fig. 2 of the main text we have therefore reconstructed the expected magnitude of the

stray field transverse to the NV axis and originating from the magnetic disc, using the expression:

$$B_{\perp,r}(\boldsymbol{\rho}, d) = \sqrt{B_y^2 + (B_z \sin(\theta_{\mathrm{NV}}) - B_x \cos(\theta_{\mathrm{NV}}))^2}. \tag{9}$$

Note that the reconstructed map of $B_{\perp,r}(\boldsymbol{\rho}, d)$ will exactly match the map extracted from the spin level mixing given knowledge of the uniform bias field, which in our case is known up to the direction of a small perpendicular component.

As we shall see, eq. (7) allows for an intuitive real-space interpretation.

We define the real-space expression for the *resolution function* $\alpha_{x,y}(d,t)$ of the in-plane magnetization as:

$$\begin{aligned}\alpha_{x,y}(d,t) &= \frac{1}{(2\pi)^2}\int_{\mathbf{k}} \frac{e^{-(d+t)k}\left(e^{tk}-1\right)}{k} e^{i\mathbf{k}\cdot\boldsymbol{\rho}} \mathrm{d}\mathbf{k} \\ &= \frac{1}{2\pi}\left(\frac{1}{\sqrt{d^2+r^2}} - \frac{1}{\sqrt{(d+t)^2+r^2}}\right).\end{aligned} \tag{10}$$

Note that if $t \ll d$ the previous resolution function can be simplified as:

$$\alpha_{x,y}(d, t \ll d) \approx \frac{1}{2\pi}\frac{dt}{(d^2+r^2)^{3/2}}. \tag{11}$$

In the same way, we define the real-space expression for the *resolution function* $\alpha_z(d,t)$ of the out-of-plane magnetization as:

$$\begin{aligned}\alpha_z(d,t) &= \frac{1}{(2\pi)^2}\int_{\mathbf{k}} \frac{e^{-(d+t)k}\left(e^{tk}-1\right)}{k^2} e^{i\mathbf{k}\cdot\boldsymbol{\rho}} \mathrm{d}\mathbf{k} \\ &= -\frac{1}{(2\pi)^2}\int_{d'}\int_{\mathbf{k}} \frac{e^{-(d'+t)k}\left(e^{tk}-1\right)}{k} e^{i\mathbf{k}\cdot\boldsymbol{\rho}} \mathrm{d}\mathbf{k}\mathrm{d}d' \\ &= -\int \alpha_{x,y}(d',t)\mathrm{d}d' = \frac{1}{2\pi}\log\left(\frac{d+t+\sqrt{(d+t)^2+r^2}}{d+\sqrt{d^2+r^2}}\right).\end{aligned} \tag{12}$$

Once more, if $t \ll d$ the previous resolution function can be simplified as:

$$\alpha_z(d, t \ll d) \approx \frac{1}{2\pi}\frac{t}{(d^2+r^2)^{1/2}}. \tag{13}$$

The spatial dependence of these resolution functions or effective *point spread functions* (PSFs) for magnetometry is plotted to the right of Supplementary Figure 2 for different sets of parameters.

With such notation, no approximations, and using the convolution theorem we can rewrite the real space expression for $\mathbf{B}(\boldsymbol{\rho}, d)$ as:

$$\mathbf{B}(\boldsymbol{\rho}, d) = -\frac{\mu_0 M_{\mathrm{s}}}{2}\begin{pmatrix} -\alpha_z(d,t) * \frac{\partial^2}{\partial x^2} & -\alpha_z(d,t) * \frac{\partial^2}{\partial y \partial x} & \alpha_{x,y}(d,t) * \frac{\partial}{\partial x} \\ -\alpha_z(d,t) * \frac{\partial^2}{\partial y \partial x} & -\alpha_z(d,t) * \frac{\partial^2}{\partial y^2} & \alpha_{x,y}(d,t) * \frac{\partial}{\partial y} \\ \alpha_{x,y}(d,t) * \frac{\partial}{\partial x} & \alpha_{x,y}(d,t) * \frac{\partial}{\partial y} & \alpha_z(d,t) * \nabla^2 \end{pmatrix}\begin{pmatrix} m_x(\boldsymbol{\rho}) \\ m_y(\boldsymbol{\rho}) \\ m_z(\boldsymbol{\rho}) \end{pmatrix}. \tag{14}$$

A single component of the stray magnetic field vector carries all the information, as all the other components are fixed given the first one. Due to the symmetry of our problem it is particularly illuminating to consider the B_z component:

$$B_z(\boldsymbol{\rho}, d) = -\frac{\mu_0 M_\mathrm{s}}{2}\left(\alpha_z(d,t) * \nabla^2 m_z(\boldsymbol{\rho}) + \alpha_{x,y}(d,t) * \nabla \cdot \mathbf{m}_{x,y}(\boldsymbol{\rho})\right), \tag{15}$$

with $\mathbf{m}_{x,y} = (m_x, m_y)$ the in-plane magnetization vector. It's clear that (15) contains convolutions and it therefore entails the non-locality of the dipolar tensor. At the same time, the two resolution functions $\alpha_{x,y}$ and α_z for the in-plane and out-of-plane component of the magnetization are not equal. An intuitive reason is given by the analogy between magnetic moments and current distributions. When the magnetization is out of plane, the stray field can be viewed as given by an effective current flowing at the boundaries of the region of constant m_z. This means that the magnetic field scales as $\sim 1/r$, being r the distance from the source. For the in-plane magnetization case the situation is instead equivalent to two current sheets above and below the magnetic film; in far field such sheets compensate each other much faster than $\sim 1/r$ and more like an isolated dipole of the form $\sim 1/r^3$. It is evident from (11) and (13) that α_z and $\alpha_{x,y}$ indeed scale as $\sim 1/r$ and as $\sim 1/r^3$ for very thin films.

Finally, in our experiments we have considered a stack of $N = 10$ magnetic thin films separated by a distance s. As the magnetization $M_\mathrm{s}\mathbf{m}$ is assumed to be constant through the film thickness, the stray field for the $N \neq 1$ case would read exactly like (15) with the difference that the PSFs are replaced by:

$$\begin{aligned}\alpha_z(d,t) &\to \alpha_{z,N}(d,t) = \sum_{\nu=0}^{N-1} \alpha_z(d + \nu \cdot s, t),\\ \alpha_{x,y}(d,t) &\to \alpha_{x,y,N}(d,t) = \sum_{\nu=0}^{N-1} \alpha_{x,y}(d + \nu \cdot s, t).\end{aligned} \tag{16}$$

Supplementary Note 3. Magnetization reconstruction in the *Bloch* and *Néel* gauge

In Supplementary Note 2, eq. (15) provided us with a real space interpretation of stray field magnetometry. Since convolutions commute with derivatives, we can reformulate the problem of reconstructing the underlying magnetization pattern from the stray field measurements starting from Gauss's equation:

$$B_z(\boldsymbol{\rho}, d) = -\nabla \cdot \mathbf{F}, \tag{17}$$

where the two-component vector field $\mathbf{F}(\boldsymbol{\rho}, d)$ plays the role of an effective electric field and the function $B_z(\boldsymbol{\rho}, d)$ describes the charge density.

The effective electric field can be written down as:

$$\mathbf{F} = \frac{\mu_0 M_\mathrm{s}}{2} \left(\alpha_z(d,t) * \nabla m_z(\boldsymbol{\rho}) + \alpha_{x,y}(d,t) * \mathbf{m}_{x,y}(\boldsymbol{\rho}) \right), \tag{18}$$

where $m_z(\boldsymbol{\rho})$ and $\mathbf{m}_{x,y}(\boldsymbol{\rho})$ play the role of an effective scalar and vector potential, respectively. A solution to eq. (17) is defined up to a divergenceless term, which in our case can be written as:

$$\mathbf{F} = -\nabla V + \nabla \times C_z \mathbf{u}_z, \tag{19}$$

where $C_z(\boldsymbol{\rho}, d)$ is an arbitrary function of space, a priori undetermined, and $\mathbf{u}_z$ a unit vector perpendicular to the surface. We choose $C_z(\boldsymbol{\rho}, d)\mathbf{u}_z$ to point in the z direction because $\mathbf{F}$ is oriented in the (x, y) plane. Our derivation diverges from classical electromagnetism (EM)[8]. In particular, in EM the curl of the vector potential is determined by a magnetic field measurement. In our effective problem we only have access to B_z meaning that $\nabla \times \mathbf{m}_{x,y}$, and in turn C_z, is fully undetermined.

Even if we had full knowledge of $\mathbf{F}$, a second degree of arbitrariness in the knowledge of the vector and scalar potential comes, as in EM, from the following *gauge*-like degree of freedom:

$$\begin{aligned} m_z(\boldsymbol{\rho}) &= m'_z(\boldsymbol{\rho}) + \Lambda, \\ \mathbf{m}_{x,y}(\boldsymbol{\rho}) &= \mathbf{m}'_{x,y}(\boldsymbol{\rho}) - \alpha_{x,y}^{-1}(d,t) * \alpha_z(d,t) * \nabla \Lambda, \end{aligned} \tag{20}$$

where $\Lambda(\boldsymbol{\rho}, d)$ is an arbitrary function of space.

As explained in the main text, in order to fix the arbitrary functions $\Lambda(\boldsymbol{\rho}, d)$ and $C_z(\boldsymbol{\rho}, d)$ and therefore classify the different spin structures producing the measured stray field, we proceed in analogy with EM. Each physically distinct configuration of the spin texture is obtained after making local assumptions about the vector field $\mathbf{m}$, with a procedure that resembles standard gauge fixing in EM[8].

Two of these possible assumptions, motivated by the spiral (cycloid) nature of Bloch (Néel) domain walls[9] and the resulting partial differential equations that need to be solved in order to determine $\mathbf{m}$ are reported in the next Sections. By a solution in the Bloch gauge to the stray field equation, we mean a solution to (17) for $\mathbf{m}$ in which we make the local assumption:

$$\nabla \cdot \mathbf{m}_{x,y} = 0, \tag{21}$$

whose physical justification has been given in the main text.

Since $\mathbf{m}_{x,y}$ plays the role of an effective vector potential, the condition (21) reminds us of the Coulomb gauge in EM. Exactly as in EM, in the Coulomb gauge the equation providing us with the scalar potential is the Poisson one:

$$-\frac{2B_z(\boldsymbol{\rho}, d)}{\mu_0 M_\mathrm{s}} = \alpha_z(d,t) * \nabla^2 m_z(\boldsymbol{\rho}), \tag{22}$$

easy to solve in Fourier space. We also know that the solution to (22) is unique once boundary conditions are fixed[8]. Once $m_z(\boldsymbol{\rho})$ is found, we can then obtain $\mathbf{m}_{x,y}$ by solving $\nabla \cdot \mathbf{m}_{x,y} = 0$. The complete partial non-linear differential equation in the azimuthal angle $\phi(\boldsymbol{\rho})$ reads as:

$$\nabla \cdot \sqrt{m_s^2(\boldsymbol{\rho}) - m_z^2(\boldsymbol{\rho})} \begin{pmatrix} \cos(\phi) \\ \sin(\phi) \end{pmatrix} = \mathscr{B}(\phi, \boldsymbol{\rho}) = 0. \tag{23}$$

Eq. (23) takes normalization to the space-dependent saturation magnetization $m_s(\boldsymbol{\rho})$ into account (see also Supplementary Note 6 for more details on this last point). We obtain a solution to (23) variationally, by minimizing the following cost function with respect to ϕ:

$$\mathscr{C}(\phi) = \int \mathscr{B}^2(\phi, \boldsymbol{\rho}) \mathrm{d}\boldsymbol{\rho}. \tag{24}$$

A very brief reminder of the popular steepest descent method we have used for minimizing the quadratic form in (24) is presented in Supplementary Note 4. By a solution in the Néel *gauge* to the stray field equation, we mean a solution to (17) for $\mathbf{m}$ in which we make the local assumption:

$$\nabla \times \mathbf{m}_{x,y} = 0. \tag{25}$$

Fixing the curl of $\mathbf{m}_{x,y}$ is equivalent to fixing the curl for the effective electric field $\mathbf{F}$ or, equivalently, the function C_z in (19). The vector field $\mathbf{F}$ becomes therefore conservative and it can be obtained explicitly from:

$$B_z(\boldsymbol{\rho}, d) = \nabla^2 V,$$
$$\mathbf{F} = -\nabla V. \tag{26}$$

At this point, an explicit solution to the stray field equation is still not possible as we retain the degree of freedom given by the arbitrary function $\Lambda(\boldsymbol{\rho}, d)$ (note that a transformation like the one in (20) preserves the curl of the vector field $\mathbf{m}_{x,y}$). In order to further reduce the manifold of possible solutions, we introduce the normalization of the vector field $\mathbf{m}$ in the form of:

$$\mathbf{F} = \frac{\mu_0 M_\mathrm{s}}{2}\left(\alpha_z(d,t) * \nabla m_z(\boldsymbol{\rho}) + \alpha_{x,y}(d,t) * \sqrt{m_s^2(\boldsymbol{\rho}) - m_z^2(\boldsymbol{\rho})}\mathbf{u}_\phi\right), \tag{27}$$

with $\mathbf{u}_\phi$ the unit vector $(\cos(\phi), \sin(\phi))$. Eq. (27) represents two coupled non-linear partial differential equations in ϕ and m_z. In order to produce Fig. 3e of the main text we have solved it by minimizing (with respect to ϕ and m_z) the following cost function, variationally:

$$\mathscr{C}(\phi, m_z) = \int \left[(F_x(\phi, m_z, \boldsymbol{\rho}) + \partial_x V)^2 + (F_y(\phi, m_z, \boldsymbol{\rho}) + \partial_y V)^2\right] \mathrm{d}\boldsymbol{\rho}. \tag{28}$$

In Supplementary Note 5 we discuss the degeneracy of the solution once normalization and curl have been fixed.

Supplementary Note 4. Steepest descent minimization

The minimization of a quadratic form using numerical, iterative steepest descent procedures is reported in several textbooks[10], for instance in the context of energy functionals.

In general, it is well known that a quadratic function $\mathscr{C}(\{x_\alpha\})$ of N-variables $x_\alpha, \alpha = 1 \dots N$ can be minimized starting from the guess $x_{\alpha,0}$, by iteratively moving antiparallel to the gradient direction, e.g.[10]:

$$x_{\alpha,i+1} = x_{\alpha,i} - \lambda \frac{\partial \mathscr{C}(\{x_\alpha\})}{\partial x_\alpha}\Big|_{x_{\alpha,i}}, \tag{29}$$

where λ is a constant and the $i = 0, \cdots N_s$-index refers to the iteration number. The previous follows from the fact that gradients are orthogonal to isolines directions:

$$\mathrm{d}\mathscr{C}(\{x_\alpha\}) = 0 = \sum_\alpha \frac{\partial \mathscr{C}}{\partial x_\alpha} \mathrm{d}x_\alpha. \tag{30}$$

When $\mathscr{C}$ becomes a functional, $N \to \infty, \{x_\alpha\} \to \phi(\alpha)$ and the functional increment upon a change $\phi(\alpha)$ to $\phi(\alpha) + \eta(\alpha)$ can be written in first order as[11]:

$$\mathrm{d}\mathscr{C}(\phi(\alpha)) = \int \mathrm{d}\alpha \frac{\delta \mathscr{C}}{\delta \phi(\alpha)} \eta(\alpha). \tag{31}$$

The analogy between (31) and (30) allows to rewrite the update in (29) in the continuous limit as[10]:

$$\phi_{i+1}(\alpha) = \phi_i(\alpha) - \lambda \frac{\delta \mathscr{C}}{\delta \phi(\alpha)}\Big|_{\phi_i(\alpha)}, \tag{32}$$

where the derivative with respect to $\mathscr{C}$ is a functional one[11]. The functionals that we have to minimize in this work have, like (24) and (28), the general form:

$$\mathscr{C}(\phi(\boldsymbol{\rho})) = \int \mathscr{L}^2(\phi, \boldsymbol{\rho}) \mathrm{d}\boldsymbol{\rho}. \tag{33}$$

The functional derivative in (32) can therefore be computed using chain derivatives as:

$$\frac{\delta \mathscr{C}}{\delta \phi(\boldsymbol{\rho})} = 2\mathscr{L}(\phi, \boldsymbol{\rho}) \frac{\delta \mathscr{L}}{\delta \phi(\boldsymbol{\rho})} - 2\nabla \mathscr{L}(\phi, \boldsymbol{\rho}) \cdot \frac{\partial \mathscr{L}}{\partial \nabla \phi(\boldsymbol{\rho})}. \tag{34}$$

We then numerically implement (32) in order to obtain the function ϕ that minimizes $\mathscr{C}$.

Supplementary Note 5. Uniqueness of the solution at fixed gauge

Fixing the curl of $\mathbf{m}_{x,y}$ and locally imposing a normalization of the ordered moment does not guarantee that the solution reproducing a given target stray field will be unique.

In this Section we first discuss which kind of transformations would preserve the curl and normalization of the vector field and will finally briefly comment on the uniqueness of the solution. As known from standard EM, a gauge transformation like (20) would preserve the curl of the magnetic structure and the field it produces. On the other hand, in general that same transformation does not preserve the normalization of the vector field. We start rewriting (20) in the following form:

$$\begin{aligned} m_{1,z} &= m_{2,z} + \Lambda, \\ \mathbf{m}_{1,x,y} &= \mathbf{m}_{2,x,y} + \nabla\Lambda'. \end{aligned} \tag{35}$$

If we want to locally have $||\mathbf{m}_1|| = ||\mathbf{m}_2||$ we find that the following must hold:

$$2\mathbf{m}_1 \cdot \begin{pmatrix} \nabla\Lambda' \\ \Lambda \end{pmatrix} = \begin{pmatrix} \nabla\Lambda' \\ \Lambda \end{pmatrix} \cdot \begin{pmatrix} \nabla\Lambda' \\ \Lambda \end{pmatrix}. \tag{36}$$

The previous equation fixes a condition for the norm of the vector $\mathbf{\Delta m} = (\nabla\Lambda', \Lambda)$. In particular, one can see that if we assume $||\mathbf{m}_1|| = ||\mathbf{m}_2|| = 1$, then $||\mathbf{\Delta m}|| = 2\cos(\theta_1 - \theta_{\mathbf{\Delta m}})$, where $\theta_1 - \theta_{\mathbf{\Delta m}}$ is the angle between the $\mathbf{m}_1$ and the $\mathbf{\Delta m}$ vector. Eq. (36) can be easily solved for Λ in special cases, which allow us to prove that in general fixing the curl of $\mathbf{m}_{x,y}$ and locally imposing a normalization of the ordered moment does not automatically guarantee a unique solution for the magnetic pattern.

Assume for instance that $\theta_1 = 0$ everywhere in space, meaning we are considering a ferromagnetic pattern, which clearly has zero curl. Eq. (36) then reduces to:

$$\alpha_z * \nabla\left(2\cos^2(\theta_{\mathbf{\Delta m}})\right) = -\alpha_{x,y} * \left(\sin(2\theta_{\mathbf{\Delta m}})\mathbf{u}_{\phi_{\mathbf{\Delta m}}}\right), \tag{37}$$

where $\mathbf{u}_\phi = (\cos(\phi_{\mathbf{\Delta m}}), \sin(\phi_{\mathbf{\Delta m}}))$ contains the azimuthal angle of the $\mathbf{\Delta m}$ vector. Finding a solution to (37) is complicated by the presence of convolutions.

We make the only assumption that the sinusoidal functions in $\theta_{\mathbf{\Delta m}}$ have a Fourier spectrum centred around $\tilde{\mathbf{k}}$, such that the convolutions can be approximated with multiplications:

$$\alpha_z(\tilde{\mathbf{k}})\nabla\left(\cos(2\theta_{\mathbf{\Delta m}})\right) = -\alpha_{x,y}(\tilde{\mathbf{k}})\left(\sin(2\theta_{\mathbf{\Delta m}})\mathbf{u}_\phi\right). \tag{38}$$

We can now evaluate the ratio $\alpha_{x,y}(\tilde{\mathbf{k}})/\alpha_z(\tilde{\mathbf{k}})$ using the results in Supplementary Note 2 and obtain:

$$\nabla\theta_{\mathbf{\Delta m}} = \frac{\tilde{k}}{2}\mathbf{u}_{\phi_{\mathbf{\Delta m}}}. \tag{39}$$

So, in essence, good solutions for $\Delta\mathbf{m}$ are those in which its polar angle has a gradient with a constant norm. Functions linear with the spatial coordinate will be solutions, such as plane waves $\theta_{\Delta\mathbf{m}} = (\tilde{\mathbf{k}} \cdot \mathbf{r})/2$ with $\mathbf{u}_{\phi_{\Delta\mathbf{m}}} \parallel \tilde{\mathbf{k}}$ or radial waves $\theta_{\Delta\mathbf{m}} = \tilde{k}r/2$. All these solutions are therefore cycloids, in the definition given in the main text.

We found that cycloids with a single wave vector and constant ordered moment can actually produce no stray field, as ferromagnetic states also do not according to (15).

On the other hand, we also note that if we fix boundary conditions, e.g. assume the magnetic moments to be in a ferromagnetic state for $r \to \infty$, then in order to avoid having $B_z \neq 0$ at the boundary between the ferromagnetic and the plane wave region we need to select $\tilde{k} \to 0$.

We therefore argue that fixing the curl of $\mathbf{m}_{x,y}$, then locally imposing a normalization and boundary conditions selects a unique solution to the stray field equation.

Supplementary Note 6. Calibrations

In our work we have carried out a reconstruction of the underlying local magnetization configuration starting from eq. (15). The unknown parameters in the equation are the film thickness t, the NV depth d and the local value of the saturation magnetization $M_\mathrm{s} \cdot m_s(\boldsymbol{\rho})$, which has been used for the reconstruction in eq. (23) and (27).

In order to calibrate these values we start from a simultaneous measurement of the magnetic disc topography and stray field map at saturation, as shown in Supplementary Figure 3. We first compare the stray field maps in Supplementary Figure 3a and Supplementary Figure 3b with the surface topography measured by monitoring the vertical movement of the tip, shown in Supplementary Figure 3c. In each image we superimpose a black dashed boundary qualitatively representing the region within which a magnetic signal is measured. By comparison of this boundary with the surface topography, we see that magnetic signal is measured from the region in the disc having a constant thickenss. We conclude that within the field of view in Supplementary Figure 3a and Supplementary Figure 3b, it is the saturation magnetization $M_\mathrm{s} \cdot m_s(\boldsymbol{\rho})$ that varies and not the film thickness t. We therefore retain t as constant in eq. (15) and make use of eq. (16) in order to compute the resolution functions. In particular, the values used during the deposition are $t = 1.1$ nm, $N = 10$, $s = 7$ nm, in agreement with the measured total thickness of the film in Supplementary Figure 3c. Note that for the NV depth d we use $d \sim 30$ nm, a value that SRIM calculations predict to be in agreement with the 18 keV implantation energy of our diamond[1].

We now assume the magnetization to be out-of-plane due to magnetic anisotropy[12] in the regime in which the skyrmion disappears; such assumption is well supported by looking at the spatially homogeneous stray field pattern for B_z measured at the magnetic disc edge in Supplementary Figure 3b. With this information we can now estimate the local value of the saturation magnetization for Co. By a direct inversion of the B_z profiles in fig:Bounda and Supplementary Figure 3b, i.e. solving eq. (22) for $M_\mathrm{s} \cdot m_z$ in both regimes, we obtain Fig. 3d of the main text. It should be pointed out that in order to obtain the inversion at saturation we solve eq. (22) and work in the Bloch gauge because in this regime $\mathbf{m}_{x,y} = 0$, which therefore satisfies $\nabla \cdot \mathbf{m}_{x,y} = 0$. The $M_\mathrm{s} \cdot m_z(\boldsymbol{\rho})$ value at 11.8 mT (saturation) is equivalent to $M_\mathrm{s} \cdot m_s(\boldsymbol{\rho})$, telling us how is the nominal M_s value locally renormalized ($m_s(\boldsymbol{\rho})$) due to variations in the saturation magnetization. With this procedure we estimate (see Fig. 3d of the main text) a maximum value for $M_\mathrm{s} \cdot m_s(\boldsymbol{\rho})$ of $M_\mathrm{s} \simeq 3.6 \cdot 10^5$ A/m at the disc centre. We then independently measured, using Vibrating Sample Magnetometry (VSM), the nominal value of M_s for our film by means of a reference Si wafer placed in the sputtering chamber together with our tip during the deposition process. We found $M_\mathrm{s} \simeq 3.8 \cdot 10^5$ A/m (see

Supplementary Figure 4), in agreement with the NV measurement. Note that such value for M_s is less than half the bulk value, suggesting a magnetic dead layer due to roughness or oxidation. In-plane (hard axis) measurements (not shown) revealed saturation fields of ≈ 5 kOe.

Based on the implantation energy of the diamond used in our experiments, NV centres in our pillars are expected to be present at an estimated depth of $d \sim$30 nm below the surface. The aim of this section is to study the stability of the reconstructed solutions upon a change of the NV-sample distance. Intuitively, as the stray field B_z is directly related to the gradient of the local magnetization, for increasing values of d the spatial variations of the local magnetization will have to increase in order for B_z to remain constant. This is what we observe, e.g., in Supplementary Figure 5a, where solutions at different values of d are obtained in the Bloch gauge for two values of the applied field. As one can see, the solutions at larger values of d in the Bloch gauge (obtained by a direct solution of a Poisson-like equation in Fourier space) are affected by more noise at high wavenumbers due to the problem of *downward propagation*[5]. In addition, we notice that at saturation the magnetization varies by only $\approx \pm 6\%$ for an NV depth change of ± 20 nm.

In order to avoid the forward propagation issue and check for the stability of the solution obtained in the Néel gauge upon a change in the NV depth, we approximate $M_\mathrm{s} \simeq 3.6 \cdot 10^5$ A/m (as shown in Fig. 3d of the main text and consistently with the magnetometry data in Supplementary Figure 4) and obtain the set of solutions in Supplementary Figure 5b. Qualitatively, we can see the skyrmion walls getting slightly sharper with larger NV distance. Regardless, we always obtain a domain-wall like solution for the skyrmion, with the same characteristic diameter $\rho_0 \approx 200$ nm. In Supplementary Figure 5c we plot the topological number as a function of the parameter λ defined in eq. (40) for different values of the parameter d. This analysis of the topology of the solution allows us to isolate the Néel configuration even when the parameter d is modified.

Supplementary Note 7. Continuous tuning of the magnetic structure

The solutions presented in Supplementary Note 3 are only two special cases of the infinitely many $\mathbf{m}$ satisfying (15) given a stray field B_z. In order to continuously explore the solution manifold, we start from the Bloch $\mathbf{m}_\mathrm{B}$ and first perform a local rotation $\bar{\mathbf{R}}_z(\Delta\phi(\lambda))\mathbf{m}_\mathrm{B}$ of the magnetic structure about the z-axis by an angle $\Delta\phi(\lambda)$, defined as:

$$\Delta\phi(\lambda) = \phi_\mathrm{B} + \lambda\left(\phi_\mathrm{N} - \phi_\mathrm{B}\right). \tag{40}$$

In (40), $\phi_\mathrm{B}, \phi_\mathrm{N}$ are the solutions for the azimuthal angles in (24) and (28) for the Bloch and Néel case, respectively, and $0 \leq \lambda \leq 1$ is a constant. As $\lambda \to 1$, the orientation of the in-plane local moments will be parallel to the one obtained in the Néel solution; however, the stray field produced by the resulting spin structure $\mathbf{m}_{\lambda,\mathrm{B}} = \bar{\mathbf{R}}_z(\Delta\phi(\lambda))\mathbf{m}_\mathrm{B}$ will not match the target field measured in experiments.

In order to preserve the in-plane orientation and match the target field, starting from $\mathbf{m}_{\lambda,\mathrm{B}}$ we locally rotate the structure about the locally varying in-plane axis $\mathbf{u}(\boldsymbol{\rho})$ perpendicular to $\mathbf{m}_{\lambda,\mathrm{B}}$ and defined as:

$$\mathbf{u} = \frac{1}{||\mathbf{m}^{x,y}_{\lambda,\mathrm{B}}||}\begin{pmatrix} m^y_{\lambda,\mathrm{B}} \\ -m^x_{\lambda,\mathrm{B}} \\ 0 \end{pmatrix}. \tag{41}$$

Rotations $\bar{\mathbf{R}}_\mathbf{u}$ by an angle $\theta(\boldsymbol{\rho})$ about the local axis $\mathbf{u} \perp \mathbf{m}_{\lambda,\mathrm{B}}$ can be readily expressed using the Rodriguez's formula[13]:

$$\bar{\mathbf{R}}_\mathbf{u} = \cos(\theta)\bar{\mathbf{I}} + \sin(\theta)[\mathbf{u}]_\times, \tag{42}$$

where $[\ldots]_\times$ is the cross product matrix and $\bar{\mathbf{I}}$ is the identity. After the $\bar{\mathbf{R}}_z(\Delta\phi(\lambda))$ and $\bar{\mathbf{R}}_\mathbf{u}$ rotation, the final structure reads as:

$$\mathbf{m}_{\theta,\lambda,\mathrm{B}} = \bar{\mathbf{R}}_\mathbf{u}\bar{\mathbf{R}}_z(\Delta\phi(\lambda))\mathbf{m}_\mathrm{B} = \cos(\theta)\mathbf{m}_{\lambda,\mathrm{B}} + \sin(\theta)\mathbf{p}_{\lambda,\mathrm{B}}, \tag{43}$$

where the vector $\mathbf{p}_{\lambda,\mathrm{B}}$ is a vector orthogonal to and with the same norm of $\mathbf{m}_{\lambda,\mathrm{B}}$, that can be written as:

$$\mathbf{p}_{\lambda,\mathrm{B}} = \begin{pmatrix} -\frac{m^z_{\lambda,\mathrm{B}} m^x_{\lambda,\mathrm{B}}}{||\mathbf{m}^{x,y}_{\lambda,\mathrm{B}}||} \\ -\frac{m^z_{\lambda,\mathrm{B}} m^y_{\lambda,\mathrm{B}}}{||\mathbf{m}^{x,y}_{\lambda,\mathrm{B}}||} \\ ||\mathbf{m}^{x,y}_{\lambda,\mathrm{B}}|| \end{pmatrix}. \tag{44}$$

If now we assume that a certain magnetic structure $\mathbf{m}$ produces a stray field $B_z(\mathbf{m})$, then a solution for $\theta(x, y)$ in (42) can be obtained by minimizing with a numerical variational analysis (see Supplementary Note 4) the following functional:

$$\mathscr{C}(\theta) = \int \left(B_z(\mathbf{m}_{\theta,\lambda,\mathrm{B}}) - B_z(\mathbf{m}_{\mathrm{B}})\right)^2 \mathrm{d}\boldsymbol{\rho}. \tag{45}$$

The resulting function θ is found to be continuous and it's used to compute the solutions in Fig. 4 of the main text.

Supplementary Note 8. Micromagnetic simulations: stability of the Néel caps

To understand the origin of the observed right-handed chirality in a material with left-handed DMI, we performed full 3D micromagnetic simulations of a skyrmion in a multilayer system with ten magnetic layers. Here, we assume a CoFeB-based system with a saturation magnetization of $M_{\rm s} = 10^6$ A/m, an exchange constant of $A = 10$ pJ/m, an anisotropy field of $\mu_0 H_k = 0.2$ T and a variable strength of the DMI D_i. Each of the ten repeats consists of 1 nm of magnetic material and 7 nm of non-magnetic spacers, such as Pt and Ta. Each repeat is simulated by a single cell in z direction by using the effective medium model[12,14]. We set a tiny inter-layer exchange couping of 0.1 pJ/m to break the degeneracy between clockwise and counterclockwise Bloch configurations. Generally, the parameters were chosen to obtain skyrmions with 50 nm radius in an out-of-plane magentic field of about 50 mT.

Supplementary Figure 6 shows the final magnetic state at different values of DMI after relaxing a skyrmion state similar to Supplementary Figure 6a for at least 20 ns in the magnetic field shown in Supplementary Figure 6f. For DMI values $D_i < 1$ mJ/m^2 (Supplementary Figure 6c) we observe a right-handed chirality in the top-most layer. The domain wall angle in each layer as a function of D_i is plotted in Supplementary Figure 6d. A domain wall angle $> 90^\circ$ indicates a right-handed wall. The lower the DMI the more layers show a right-handed chirality. The layer number in which the chiralty switches from left-handed to right-handed (i.e., the layer in which the configuration is purely Bloch-like) is plotted in Supplementary Figure 6e as a function of D_i. It is important to note that, in contrast to other surface-sensitive techniques, such as Photo-Emission Electron Microscropy (PEEM), NV magnetometry can distinguish beween the various cases shown in Supplementary Figure 6d even though the top layer magnetization remains the same.

For a uniform magnetization along z, the field required to stabilize a skyrmion of a given size ($R = 50$ nm in the present case) is proportional to D_i. This behavior is confirmed in the high DMI regime in Supplementary Figure 6f, i.e., for DMI values where all layers have the same chirality. The formation of flux-closure domains, however, breaks this trend. Specifically, the stabilizing field of 26 mT for the zero DMI case could be misinterpreted as a DMI strength of $D_i \approx 0.5$ mJ/m^2 if a uniform magnetization along z is assumed (see red line in Supplementary Figure 6f). This observation underlines the significance of volume stray field interactions and flux closure domains for the interpretation and design of skyrmions in magnetic multilayers.

Supplementary Note 9. Global versus local effective gauge fixing

In Supplementary Note 3 an effective gauge was imposed in order to find a solution for the local magnetization. Conditions such as those in eq. (21) and (25) were imposed *globally* through the magnetic stack, meaning that the magnetization pattern $\mathbf{m}(\boldsymbol{\rho})$, the pattern's helicity and chirality were the same regardless the magnetic layer considered.

In the main text we compare our experimental data with a model in which the top and bottom three layers have opposite Néel chirality, contrary to the Bloch-like intermediate layers. This case can be easily considered starting from eq. (16), introducing parameters c_i such that now:

$$\alpha_{x,y}(d,t) \to \alpha_{x,y,N}(d,t) = \sum_{\nu=0}^{N-1} c_\nu \alpha_{x,y}(d + \nu \cdot s, t). \tag{46}$$

Opposite chirality between, say, layer i and j can be imposed by simply setting $c_i = -c_j$. This results in a new $\alpha_{x,y}(d,t)$. In this way, the magnetization pattern $\mathbf{m}(\boldsymbol{\rho})$ can still be considered as layer-independent but the in-plane magnetization will be summed up oppositely between the layers i and j; within the minimization process, this effectively inverts the relative chirality between the layers. In order to account for the intermediate layers hosting Bloch-like skyrmions, we have decided to run the minimization process still within the Néel gauge, but setting for the intermediate i-layer the coefficients $c_i = 0$. This condition implies that the term $\nabla \cdot \mathbf{m}_{x,y}$ will not contribute, for those layers, to B_z, as it should be for a real Bloch solution. The last method allows us to obtain a layer-independent m_z profile, which we plot in Fig. 5c of the main text.

Supplementary References.

[1] Spinicelli, P. *et al.*, Engineered arrays of nitrogen-vacancy colour centres in diamond based on implantation of CN^- molecules through nanoapertures. New Journ. of Phys. **13**, 025014 (2011).

[2] Gali, A., Fyta, M., & Kaxiras, E. Ab initio supercell calculations on nitrogen-vacancy center in diamond: Electronic structure and hyperfine tensors. Phys. Rev. B **77**, 155206 (2008).

[3] Balasubramanian, G. *et al.*, Nanoscale imaging magnetometry with diamond spins under ambient conditions. Nature **455**, 648 (2008).

[4] van der Sar, T., Casola, F., Walsworth, R., & Yacoby, A. Nanometre-scale probing of spin waves using single electron spins. Nature Comm. **6**, 7886 (2015).

[5] Blakely, R. J. Potential theory in gravity and magnetic applications. (Cambridge University Press, Cambridge, 1996).

[6] Dreyer, S. *et al.*, Quantitative imaging of stray fields and magnetization distributions in hard magnetic element arrays. Journ. Appl. Phys. **101**, 083905 (2007).

[7] Lima, A. E., & Weiss, B. P, Obtaining vector magnetic field maps from single-component measurements of geological samples. Journ. of Geo. Research **114**, B06102 (2009).

[8] Griffiths, D. J., Introduction to Electrodynamics. Upper Saddle River, N.J: Prentice Hall (1999).

[9] Tetienne, J.P. *et al.*, The nature of domain walls in ultrathin ferromagnets revealed by scanning nanomagnetometry. Nature Comm. **6**, 6733 (2015).

[10] see e.g. Martin, R. M., Electronic Structure: Basic Theory and Practical Methods. (Cambridge University Press, Cambridge, 2004).

[11] see e.g. Binney, J. J., Dowrick, N. J., Fisher, A. J. and Newman, M. E. J., The Theory of Critical Phenomena, An Introduction to the Renormalization Group. (Clarendon Press, Oxford, 1992).

[12] Woo, S. *et al.*, Observation of room-temperature magnetic skyrmions and their current-driven dynamics in ultrathin metallic ferromagnets. Nature Mater. **15**, 501-506 (2016).

[13] see e.g. Szeliski, R., Computer Vision: Algorithms and Applications. (Springer-Verlag, London, 2011).

[14] Lemesh, I., Büttner, F. and Beach, G. S. D. Accurate model of the stripe domain phase of perpendicularly magnetized multilayers. Phys. Rev. B **95**, 174423 (2017).
